# Effective phage cocktail to combat the rising incidence of extensively drug-resistant *Klebsiella pneumoniae* sequence type 16

**DOI:** 10.1080/22221751.2022.2051752

**Published:** 2022-04-07

**Authors:** Willames M. B. S. Martins, Mei Li, Kirsty Sands, Michael H. Lenzi, Edward Portal, Jordan Mathias, Priscila P. Dantas, Roberta Migliavacca, James R. Hunter, Eduardo A. Medeiros, Ana C. Gales, Mark A. Toleman

**Affiliations:** a Department of Medical Microbiology, Division of Infection and Immunity, Cardiff University, Cardiff, UK; b Division of Infectious Diseases, Department of Internal Medicine, Escola Paulista de Medicina/Universidade Federal de São Paulo - UNIFESP, São Paulo, Brazil; c Department of Zoology, University of Oxford, Oxford, UK; d Division of Infection Control and Hospital Epidemiology, Hospital São Paulo, Universidade Federal de São Paulo, São Paulo, Brazil; e Department of Clinical-Surgical, Diagnostic and Pediatric Sciences, Unit of Microbiology and Clinical Microbiology, University of Pavia, Pavia, Italy

**Keywords:** Antimicrobial resistance, viruses, synergism, biofilm, enterobacterales

## Abstract

Bacteriophages are the most abundant organisms on Earth. As there are few effective treatment options against some pathogens, the interest in the bacteriophage control of multi-drug-resistant bacterial pathogens is escalating, especially for *Klebsiella pneumoniae*. This study aimed to develop a phage-based solution to the rising incidence of extensively drug-resistant clinical *Klebsiella pneumoniae* sequence type (ST16) infections starting from a set of phages recently characterized against this lineage. A phage-cocktail (Katrice-16) composed of eight lytic phages was characterized for potential use in humans. *In vitro* and *in vivo* broth inhibition and *Galleria mellonella* rescue assays were used to demonstrate the efficacy of this approach using a collection of 56 strains of *K. pneumoniae* ST16, with distinct genetic backgrounds that were collected from clinical infections from four disparate nations. Additionally, Katrice-16 anti-biofilm activity, synergism with meropenem, and activity in human body fluids were also assessed. Katrice-16 was highly active *in vitro* against our *K. pneumoniae* ST16 collection (AUC% median = 86.48%; Q1 = 83.8%; Q2 = 96.85%; Q3 = 98.85%). It additionally demonstrated excellent *in vivo* activity in *G. mellonella* rescue assays, even with larvae infected by isolates that exhibited moderate *in vitro* inhibition. We measured significant anti-biofilm activity over 12 h (*p* = .0113) and synergic activity with meropenem. In addition, we also demonstrate that Katrice-16 maintained high activity in human body fluids. Our results indicate that our cocktail will likely be an effective solution for human infections with this increasingly prevalent and often highly resistant bacterial clone.

## Introduction

The World Health Organization has stated that the expansion of *K. pneumoniae* resistant to carbapenems and third-generation cephalosporins is of great concern and is a high priority for the development of new antimicrobial agents and novel therapeutic approaches [1]. Several lineages of antibiotic-resistant *K. pneumoniae* (e.g. ST11, ST15, ST16, ST258, and ST340) are geographically widespread and represent a serious public health issue [2,3]. *K. pneumoniae* ST16 is an emergent extremely drug resistant (XDR) lineage that has been reported in several countries and is a particular concern in Brazil, where the expansion of this lineage is causing a large burden of human infection [4–9]. Specifically, the common occurrence of horizontally acquired resistance mechanisms such as KPC and NDM together with chromosomal *mgrB* inactivation that produces colistin resistance in this lineage severely limits therapeutic options and leads to poor prognosis [9].

The limited therapeutic options are currently driving the development of several alternative therapies, including bacteriophages, blue light, nanoparticles, or small peptides [10]. Bacteriophages, viruses that infect and lyse bacteria cells, represent a particularly promising tool. These viruses were initially discovered early in the twentieth century, but their clinical capacity has generally been overlooked in the Western world. However, their utility has increased with the emergence of antimicrobial resistance being especially linked to specific lineages [11,12].

Herein, we developed and tested the *in vitro* and *in vivo* efficacy of a bacteriophage cocktail with important antibacterial and anti-biofilm properties against *K. pneumoniae* ST16.

## Material and methods

### General features of the bacterial collection

A collection of 56 *Klebsiella pneumoniae* clinical isolates carrying multiple antimicrobial resistance genes (Table S1) and belonging to ST16 was assembled: 29 previously described in recent studies from Brazil (*n* = 19) [9] and Italy (*n* = 10) [8] together with 27 novel isolations from Brazil (*n* = 25), Bangladesh (*n* = 1), and Vietnam (*n* = 1). Susceptibility profiles were determined by disk-diffusion experiments with 16 antibiotics using EUCAST guidelines (Table S1). Broth microdilution and agar dilution were performed to determine the colistin and fosfomycin susceptibility, respectively [13]. The genetic relationship among these isolates was determined by Pulsed Field Gel Electrophoresis after macro-restriction using SpeI-endonuclease and interpreted by the Tenover criteria [14,15].

### Bacteriophage cocktail preparation and in vitro killing experiments

Bacteriophages were previously characterized for their genomic and microbiological properties [16], and equal volumes of eight phages (PWKp1, PWKp3, PWKp5, PWKp7, PWKp9B, PWKp14, PWKp15, and PWKp17) at 1 ∼ 3 × 10^9^ PFU/mL, were mixed resulting in a cocktail, Katrice-16, and stored at 4 °C. The general features of each phage present in Katrice-16 are detailed in Table S2.

In order to evaluate the *in vitro* lytic ability of the cocktail against 56 *K. pneumoniae* ST16, inhibition experiments were performed: Briefly, 180 μL of an OD ∼ 0.1 (10^7^ *K. pneumoniae* cells) culture was transferred to 96-well flat plates and mixed with 20 μL of Katrice-16 at 10^9^ PFU/mL resulting in a final MOI of 10. Control samples were added for each isolate (incubation of bacteria plus 20 μL of SM Buffer) for comparative purposes. Optical density (OD600) was measured every 30 min for 12 h with shaking at 200 rpm using a FLUOstar Omega microplate reader (BMG LABTECH Ltd., Aylesbury, UK). Experiments were conducted in triplicates, and inhibition curves were prepared using the average of triplicates with GraphPad Prism (v.8.4.3).

For those isolates poorly inhibited by Katrice-16 MOI 10, we also evaluated different phage/bacteria concentrations on activity. These experiments were conducted as described above, with minor alterations in the bacterial inoculum (start concentration at 10^6 ^CFU/mL), followed by increasing Katrice-16 concentration to reach MOIs 10, 100, and 1000 over 16 h of incubation.

### Synergism analysis between Katrice-16 and meropenem

Three representative isolates (KL29, OXA-232-producer; BKBR, NDM-1-producer; P28, KPC-2-producer) were selected based on their high level of carbapenem resistance and their inhibition by Katrice-16 MOI 10. Synergy experiments were performed as described by Liu and colleagues [17] with minor modifications to adjust the methodology to the antibiotic susceptibility test recommended by EUCAST/CLSI. 96-well plates were made by combination of 50 μL different meropenem concentrations (0.25–128 mg/L) and 50 μL of Katrice-16 (10^3^–10^9^ PFU/mL) both diluted on cation-adjusted Mueller-Hinton Broth and inoculated to reach a final bacterial concentration of 5 × 10^5^ CFU/mL. The plates were incubated at 37 °C under agitation (200 rpm) and the OD_600_ was measured every 30 min for 20 h on a FLUOstar Omega microplate reader. The experiments were performed in biological triplicates, and the reduction of bacterial growth was assessed by a mean of these triplicates. Synograms were generated at two-point times (10 and 20 h) based on the deduction of treated wells from the positive control (no treatment) to yield percent reduction: Reduction (%) [(OD_growthcontrol_ − OD_treatment_)/OD_growthcontrol_]  × 100 [17].

### Evaluation of in vivo efficacy of Katrice-16 using *Galleria mellonella* assay

The efficacy of the phage cocktail to rescue fatal infection was evaluated by *Galleria mellonella* assays as previously described [18] using six *K. pneumoniae* ST16 strains. We divided the larvae into five major groups: (10 larvae weighing 200–250 mg per each group) based on inoculation in each experiment: (i) *control group 1*, 10 μL of SM Buffer or 1× PBS; (ii) *control group 2*, 10 μL of Katrice-16; (iii) *infection group*, *5* μL of bacteria suspension (10^6^ CFU/mL) + 5 μL of SM Buffer; (iv) *treatment group 1*, 5 μL of bacteria suspension (10^6^ CFU/mL) + 5 μL of Katrice-16 (10^7^ PFU/mL); (v) *treatment group 2*, 5 μL of bacteria suspension (10^6^ CFU/mL) + 5 μL of Katrice-16 (10^8^ PFU/mL). All experiments were performed in triplicates.

The bacterial isolates were grown in LB broth for 3 h, centrifuged at 12,000*g* for 2 min, and washed three times with 1× PBS. Pellets were resuspended in 1× PBS, adjusted to the OD_600_ = 0.25 ± 0.01, and diluted to reach a final concentration of 10^6^ CFU/mL. Five microliters of bacteria suspension (∼ 5 × 10^3^ CFU) was injected into the last left-side proleg of the larvae using a 10 μL Hamilton syringe (701RN; Hamilton Bonaduz AG), and 5 μL of Katrice-16 (∼ 5  × 10^4^ and 10^5^ PFU), was injected into the last right-side proleg at one-hour post-infection. After treatment, larvae were incubated in the dark at 37 °C and their survival status checked at 24, 48, and 72 h.

### Katrice-16 anti-biofilm activity

Katrice-16 activity against mature biofilms produced by 30 randomly selected *K. pneumoniae* ST16 isolates was assessed. Biofilms were grown on Tryptic soy broth (TSB) supplemented with 1% glucose for 48 h with change of bacterial media after 24 h. After two days of biofilm growth, two groups were created: (i) an untreated group, that received 200 μL of TSB broth with 1% glucose; (ii) and a treated group, which received 10^8^ PFU/mL of Katrice-16 diluted in 200 μL of TSB broth with 1% glucose. We conducted the treatment over 12 and 24 h; After treatment, the plates were washed with PBS 1X, stained with crystal violet (0.2%) for 30 min and eluted in 30% (vol/vol) glacial acetic acid, as previously described [18]. The absorbance of each well was measured at 595 nm and each experiment was conducted in triplicate.

### Bacteriophage cocktail activity in human body fluids

Urine from five (male *n* = 4 and female *n* = 1) healthy donors was collected, warmed to 37 °C and tested against bacteria (uroculture) with pH verification. The samples were pooled, filtered through a 0.22 μM membrane and stored at −20 °C. Prior to each test, urine samples were defrosted and kept for 4 h at 37 °C with agitation every hour. To simulate a urinary tract infection, where the pH is generally more acidic, we used HCl 1M to adjust the pH of the second urine sample. Two urine pools were tested: (i) the original urine pool, pH 6.5 and (ii) a urine pool at pH 5.5. Katrice-16 was diluted in the urine samples, reaching a final concentration of ∼5 × 10^8^ PFU/mL and its activity was checked by comparing the bacterial growth in the presence/absence of phage cocktail. For urine inhibition tests, 180 μL of Katrice-16 diluted in urine was transferred into 96-well flat plates and mixed with 20 μL of bacterial inoculum in LB broth, resulting in a final OD_600_ of ∼0.1 (∼10^7^ CFU/mL). Thus, all experiments were carried out having 10% LB broth. Control samples were added for each isolate (incubation of 180 μL urine sample, without bacteriophage plus 20 μL of bacteria inoculum) for comparative purposes. Optical density (OD_600_) was measured every 30 min interval over 12 h with shaking at 200 rpm using FLUOstar Omega microplate reader (BMG LABTECH Ltd., Aylesbury, UK). These experiments were performed in triplicate, and the results were analysed and interpreted using GraphPad Prism (v.8.4.3).

Pooled, pre-inactivated human AB serum was used to check Katrice-16 stability and activity against the same isolates used in the urine analysis. Once thawed, the serum was kept for 1 h at 37 °C and then mixed with Katrice-16 at a final concentration of ∼5 × 10^8^ PFU/mL. For inhibition tests, 180 μL of Katrice-16 diluted in human serum was transferred into 96-well flat plates and mixed with 20 μL of bacterial inoculum in LB broth, resulting in a final OD600 of ∼0.1 (∼10^7^ CFU/mL). Control samples were added for each isolate (incubation of 180 μL human serum, without bacteriophage plus 20 μL of bacteria inoculum) for comparative purposes. The measurement of results and interpretation were performed as described above.

### Statistical analysis

Statistical analyses were performed using GraphPad Prism software (version 8.4.3, San Diego, CA, USA). One-way ANOVA followed by multiple comparison tests were used to compare AUC% and Katrice-16 activity in different human body fluids. Multiple comparison tests applied in each analysis are described in their respective figure legends. Differences in biofilm removal were evaluated by Two-tailed *t* tests. Log-rank (Mantel–Cox) test was used for survival curves rescue experiments. *P*-values of <.05 were considered statically significant.

## Results

### *In vitro* activity of Katrice-16 against XDR *K. pneumoniae* ST16 collection

**Figure 1.**
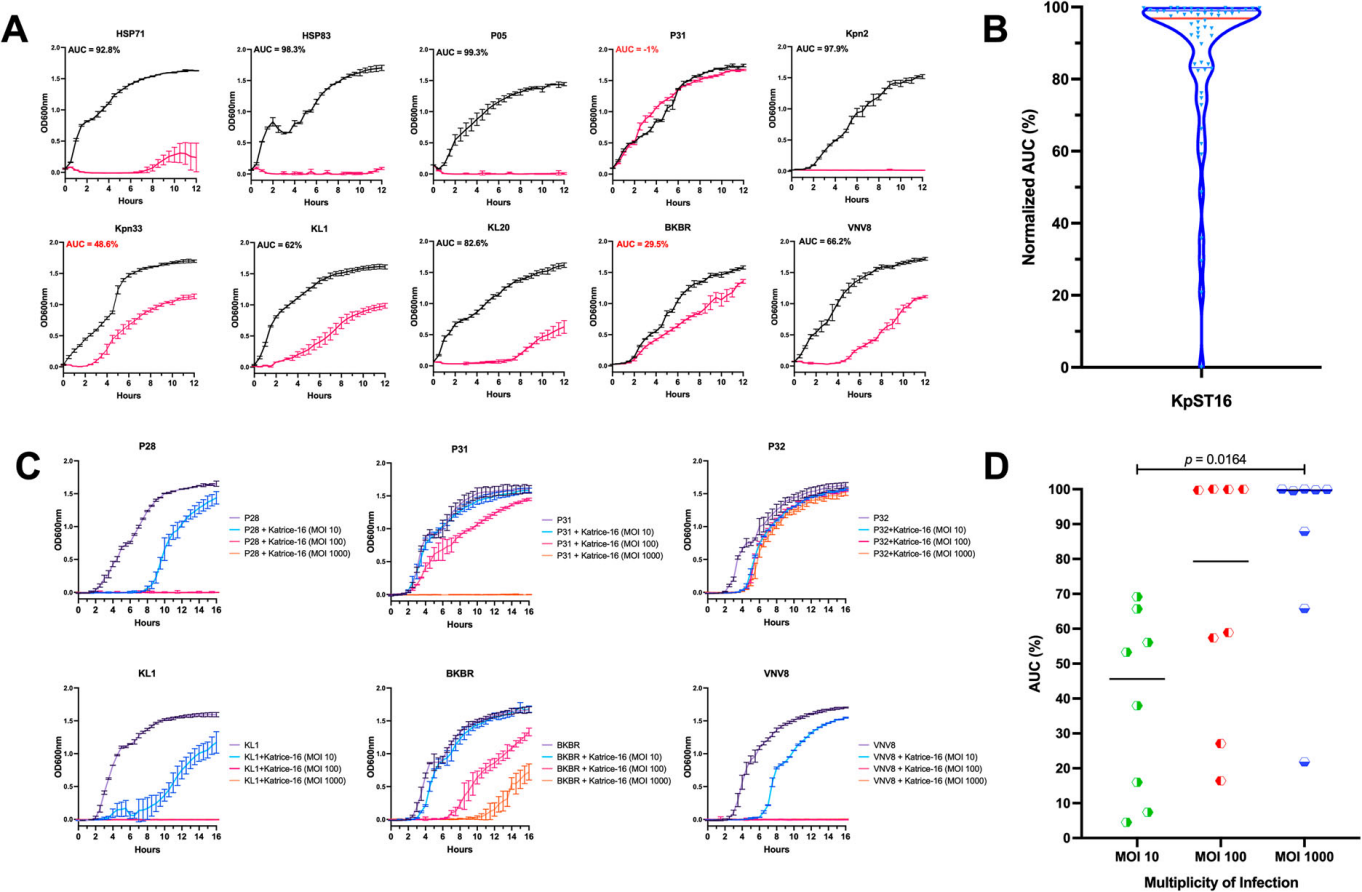
Inhibition activity of Katrice-16 against *K. pneumoniae* ST16. (A) Representative broth inhibition assays of Katrice-16 MOI 10 and *K. penumoniae* ST16 over 12 h, error bars represent the SD (*n* = 3). For each graph, the area under curve (AUC%) was calculated based on the bacterial growth in the presence/absence of Katrice-16 MOI 10. AUC% below 50% are highlighted in red. The set with the inhibition curves of all *K. pneumoniae* ST16 isolates can been seen in Figure S1. (B) Violin plot demonstrating the distribution of all 56 *K. pneumoniae* ST16 AUC% using Katrice-16 MOI 10. The blue squares are individual values of AUC%, the red line represents the mean, and the blue lines the quartiles (Q1, Q2, and Q3). (C) Broth inhibition assays of representative isolates poorly inhibited by Katrice-16 MOI 10. During this new assay, an increased concentration of Katrice-16 was used to achieve higher MOIs (100 and 1000). The experiment was conducted over 16 h and error bars represent the SD (*n* = 3). D Individual AUC% values of the eight poorly inhibited isolates submitted to a new broth inhibition test using higher MOIs. Black lines represent the median for each group and *p* value was calculated by one-way analysis of variance (ANOVA) followed by Tukey multiple comparison post hoc test [MOI 10 vs. MOI 100, *p* = .1185; MOI 10 vs. MOI 1000, *p* = .0164; MOI 100 vs. MOI 1000, *p* = .6085].

Katrice-16 demonstrated excellent *in vitro* activity against the *K. pneumoniae* ST16 collection, inhibiting bacterial growth of 98% of bacterial isolates from 12 h (Figure 1(A)). The distribution of all AUCs% (Figure 1(B)) was high, indicating effective lysis (AUC% median = 86.48%; Q1 = 83.8%; Q2 = 96.85%; Q3 = 98.85%), especially for those isolates recovered from Brazil (AUC% median = 89.62) and Italy (AUC% median = 80.39). The two Asian isolates evaluated had a non-satisfactory inhibition (BKBR, AUC% = 29.5) and a moderate inhibition (VNV8, AUC% = 66.2) (Figure 1(A); Figure S1).

We also noticed that increasing the concentration of Katrice-16 could dramatically improve the cocktail inhibition. All strains demonstrated an increase of inhibition as higher MOI were used (MOI 100 and MOI 1000) with the sole exception of isolate P32. For some isolates (e.g. P28, P31, and KL1), this increase resulted in a complete inhibition of bacterial growth, demonstrating that the relationship between the number of phages per bacterium can be a critical point for the outcome of the inhibition of bacterial growth (Figure 1(C)). Tukey’s test revealed a significant difference between tests using MOI 10 and MOI 1000 (*p* = .0164) (Figure 1(D)).

### In vivo Katrice-16 therapy rescues *G. melonella* from infection

**Figure 2.**
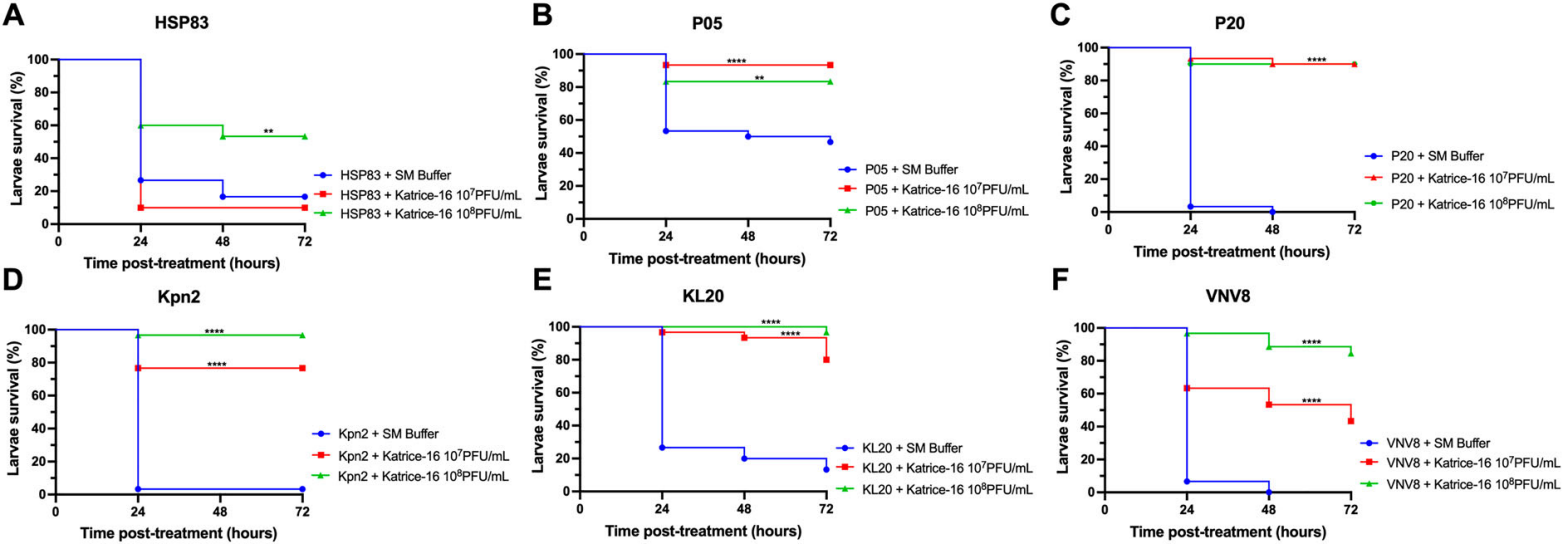
*In vivo* efficacy of Katrice-16 demonstrated by Kaplan–Meier plots showing the percent of *G. mellonella* survival after phage cocktail treatment. *P* values were calculated using Log-rank (Mantel-Cox) test for (A) (10^7^ PFU/mL, *p* = .3114, *χ*² = 1.025, d.f = 1; 10⁸ PFU/mL, *p* = .0027, *χ*² = 8.997, d.f = 1), (B) (10⁷ PFU/mL, *p* < .0001, *χ*² = 15.43, d.f = 1; 10⁸ PFU/mL, *p* = .0032, *χ*² = 8.716, d.f = 1), (C) (10⁷ PFU/mL, *p* < .0001, *χ*² = 53.76, d.f = 1; 10⁸ PFU/mL, *p* < .0001, *χ*² = 50.90, d.f = 1), (D) (10⁷ PFU/mL, *p* < .0001, *χ*² = 33.05, d.f = 1; 10⁸ PFU/mL, *p* < .0001, *χ*² = 51.40, d.f = 1), (E) (10⁷ PFU/mL, *p* < .0001, *χ*² = 33.91, d.f = 1; 10⁸ PFU/mL, *p* < .0001, *χ*² = 46.54, d.f = 1), (F) (10⁷ PFU/mL, *p* < .0001, *χ*² = 26.34, d.f = 1; 10⁸ PFU/mL, *p* < .0001, *χ*² = 56.86, d.f = 1). *indicates .01 < *p* value < .05, **indicates .001 < *p* value < .01, ***indicates .0001 < *p* value < .001, ****indicates *p* value < .0001.

Using a single Katrice-16 cocktail injection (1-hour after infection) as treatment had a dramatic impact on larvae survival for all evaluated isolates (Figure 2). Larvae survival rates after treatment with 10^7^ and 10^8^ PFU/mL of Katrice-16 increased from 0% to 90% (*p* < .0001 for both cocktail concentrations) for larvae infected with P20. This was also seen for Kpn2, increasing survival rates from 3.3% to 76.6% (*p* < .0001, 10^7^ PFU/mL) and 96.6% (*p* < .0001, 10^8^ PFU/mL) (Figure 2). The concentration of Katrice-16 was also important for some experimental outcomes. Whist no or a slight difference was observed between the two cocktail concentrations for P05, P20, and KL20, the use of a higher concentration of Katrice-16 resulted in better outcomes for the remaining strains (Figure 2). The controls used in this study indicated that Katrice-16, PBS or SM buffer had no adverse effects on the larvae (Figure S2).

### Katrice-16 impact on bacterial growth when associated with meropenem and its anti-biofilm activity

**Figure 3.**
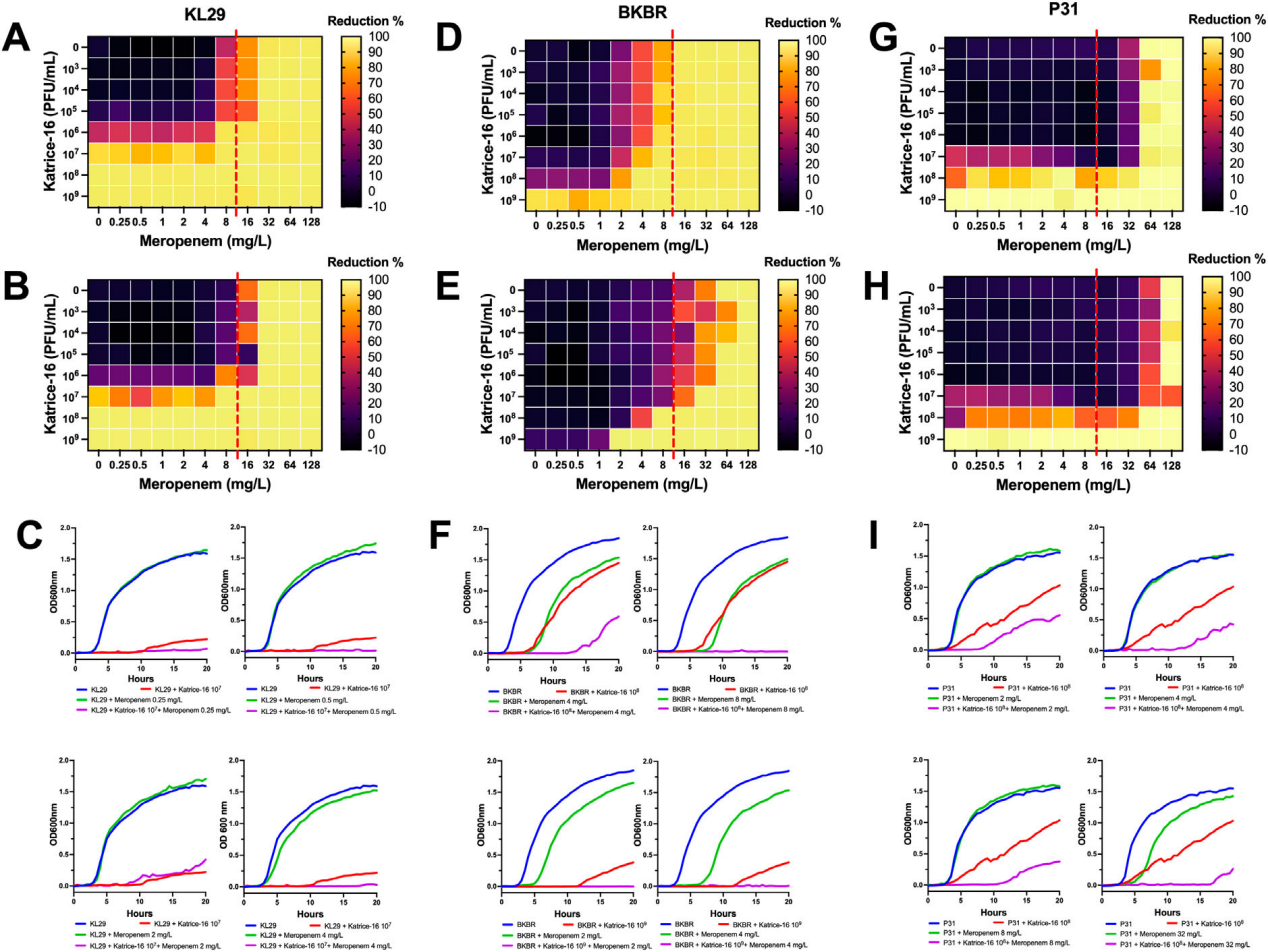
Synergism activity between Katrice-16 and meropenem against three carbapenemase-producing *K. pneumoniae* ST16 isolates. Synograms were constructed based on the data of bacterial growth in the presence/absence of antibiotic and phages over 10 and 20 h. Dashed red lines represent the susceptibility breakpoint of meropenem according to EUCAST, 2021. KL29 synograms from 10 h (A), and 20 h (B) followed by a set of four selected growth curves (C), demonstrating the effect of different concentrations of meropenem and Katrice-16 in the bacteria growth. BKBR synograms from 10 h (D), and 20 h (E) followed by a set of four selected growth curves (F), demonstrating the effect of different concentrations of meropenem and Katrice-16 in the bacteria growth. P31 synograms from 10 h (G), and 20 h (H) followed by a set of four selected growth curves (I), demonstrating the effect of different concentrations of meropenem and Katrice-16 in the bacterial growth.

The OXA-232-producing *K. pneumoniae* ST16 (KL29) showed an initial meropenem MIC of 32 mg/L and did not show any synergism when sub-inhibitory concentrations of meropenem were combined with Katrice-16 ranging from 10^3^–10^6^ PFU/mL (Figure 3). However, a significant reduction in bacterial growth was observed when 10^7^ PFU/mL was combined with sub-inhibitory concentrations of meropenem (0.25–8 mg/L), reducing the original MIC to 8 mg/mL. Synergistic results were also obtained for NDM-1-producing *K. pneumoniae* ST16 (BKBR), dropping the original MIC from 64 to 2 mg/L when associated with 10^9 ^PFU/mL of Katrice-16 (Figure 3). Among the three tested isolates, BKBR showed the highest variation between 10 and 20 h of analysis and the combination of sub-inhibitory concentrations of meropenem (2, 4, and 8 mg/L) where 10^9^ PFU/mL. P31 synergism resulted in a subtle drop of meropenem MIC (128–64 mg/mL) when combined with 10^8^ PFU/mL of Katrice-16. Although less significant than the BKBR synergism, the association of sub-inhibitory concentrations and 10^8^ PFU/mL of Katrice-16 resulted in a reduction of bacterial growth by 70–80% for 2, 4, and 32 mg/mL of meropenem, demonstrating the potential clinical impact of combination therapy.

**Figure 4.**
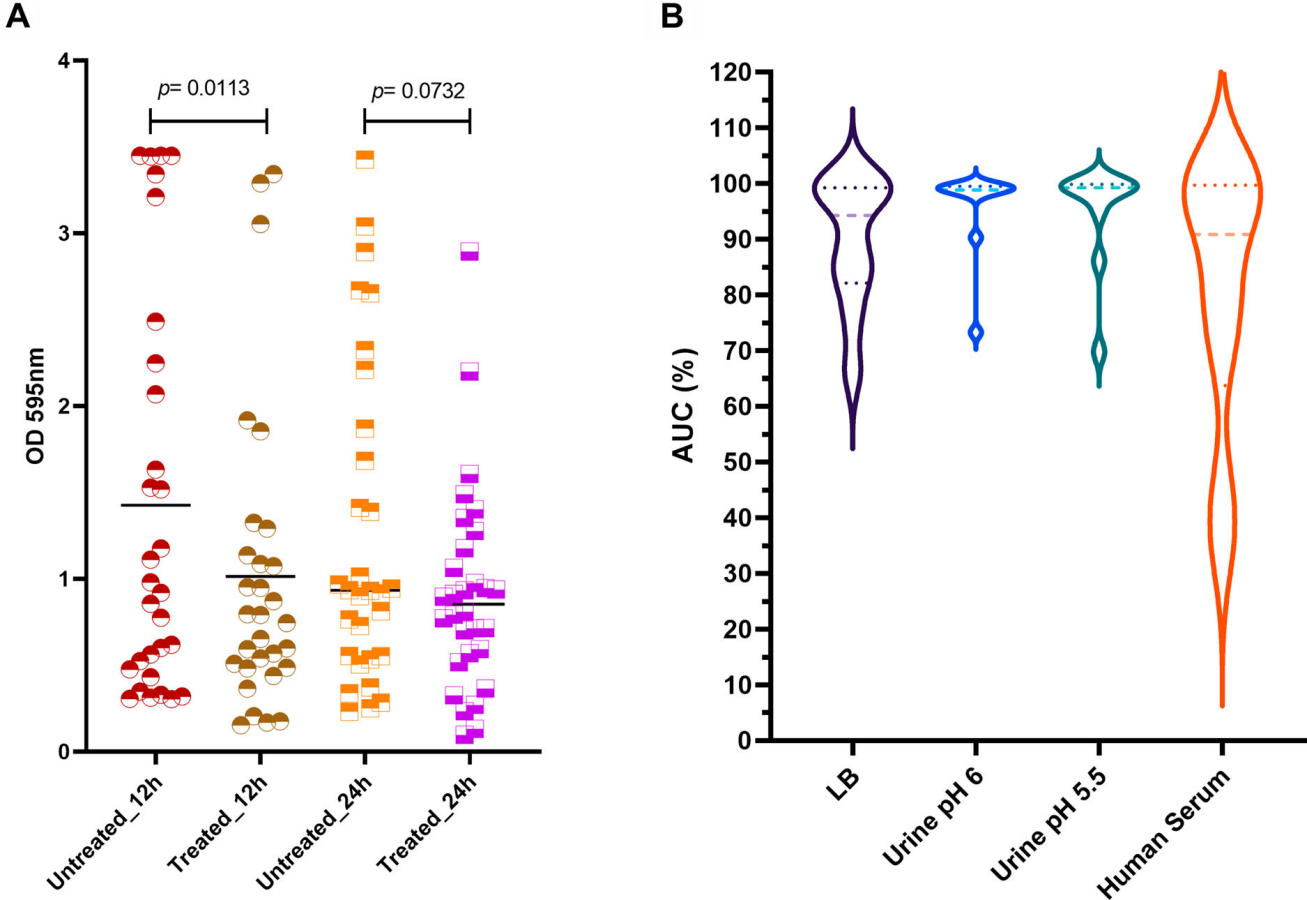
Katrice-16 antibiofilm activity and its performance in human body fluids. (A) Biofilm production of 30 *K. pneumoniae* ST16 isolates with and without treatment (12 and 24 h). Black horizontal lines represent the median of each group and *p* value was calculated by Wilcoxon signed-ranks test (12 h, *p* = .0113; 24 h, *p* = .0732). (B) Katrice-16 activity demonstrated by AUC% values of 10 selected *K. pneumoniae* ST16 in Lysogeny Broth (LB) and human body fluids (urine and human serum). Violin plots’ internal lines represent median and quartiles, and *p* values were calculated using non-parametric one-way ANOVA (Tukey’s multiple comparisons tests).

Katrice-16 was also able to combat the mature biofilm produced by *K. pneumoniae* ST16 isolates (Figure 4(A)). The median biofilm production of the 12 h-treated group was lower than 12 h-untreated group using the cocktail at 10^8^ PFU/mL, suggesting an efficient biofilm reduction (Wilcoxon test, *p* = .0113). Interestingly, the results were more promising for those isolates with higher biofilm production. In the untreated group, nine isolates had values of OD_595nm_ higher than 2, while after 12-hour treatment, only three isolates remained with values above 2. On the other hand, although the average of the 24 h-treated group was lower than 24 h-untreated group, no statistical difference was observed between the groups (Wilcoxon test, *p* = .0732).

### Activity of Katrice-16 in different human body fluids

Katrice-16 lytic activity was also evaluated in urine and human serum. Urine inhibition assays demonstrated good activity of Katrice-16, with no statistical difference in the results of inhibition obtained with both urine samples at pH 6.5 (*p* = .8052) and at pH 5.5 (*p* = 0.8676) as compared to tests performed in LB broth by Tukey's multiple comparison test. The reduction of urine pH did not have any effect on Katrice-16 activity since results of AUC% were identical (*p* = .9992). Effective Katrice-16 activity was also conserved in human serum, with no significant difference when compared to LB broth (*p* = 0.5483). There was a small AUC% median decrease in comparison to urine tests (AUC% = 80.71 of human serum vs. AUC% = 95.63 and 94.76 of urine pH 5.5 and pH 6.5, respectively). However, this difference was not statistically relevant, as observed by Tukey’s test (Figure 4(B)).

## Discussion

Katrice-16 is a phage cocktail approach developed to combat *K. pneumoniae* ST16 isolates of different genetic backgrounds. The cocktail was designed by mixing eight *Klebsiella* phages belonging to three different phage families. Although experiments to characterize the infection pathway of each bacteriophage are underway, it is likely that the different families of bacteriophages target different bacterial receptors and as such limit the emergence of phage-resistant mutants. Due to host-range results, phages belonging to the same family were also added to the cocktail. We tested different phage combinations to significantly create the best cocktail able to inhibit our tested collection. Our cocktail demonstrated excellent inhibition of the growth of the majority of XDR *K. pneumoniae* ST16 strains isolated from several disparate countries. This was demonstrated by both *in vitro* growth inhibition assays and by *in vivo* tests using the *G. mellonella* model (Figures 1(B) and Figure 2). Although some isolates were genetically related by PFGE (Table S1), they varied in phage susceptibility (Figure S1). Point mutations on phage receptors, alteration of receptor expression or capsule production can modify the phage susceptibility exhibited by a strain [19,20]. Thus, we suggested that we should not only rely on the PFGE clonal relationship of different strains to predict phage susceptibility. The rapid emergence and dissemination of antimicrobial resistance determinants have led to difficulties in the treatment of human infection and calls for alternative approaches. This is especially important since antibiotic use itself is a driver of both emergence and dissemination of resistance [21]. Successful *in vitro* and *in vivo* use of bacteriophages has been widely demonstrated in recent years [22–24], and detailed studies such as this one are necessary to ensure the robustness of bacteriophage use. Our study is important as it demonstrates that it is possible to develop a general cocktail capable of efficiently combating different isolates belonging to the same lineage from different countries. This is of major importance since it is now recognized that antibiotic resistance mechanisms are very often associated with a small number of prevalent STs of each species [25].

*In vivo* experiments using the *G. mellonella* assay also demonstrated the efficacy and safety of our cocktail. Our experiments establish that the cocktail has an impressive ability to rescue fatal infections. This is especially demonstrated for those isolates with lower AUC% values such as KL20 and VNV8 (Figure 2). This data is also important because it reveals that an excellent *in vitro* inhibition is not an absolute requirement for a satisfactory *in vivo* activity. Recent studies have also demonstrated the therapeutic efficacy of bacteriophage cocktails against several clinically relevant micro-organisms including *K. pneumoniae*, *Pseudomonas aeruginosa* and *A. baumannii* and demonstrates the utility and reproducibility of using bacteriophages to rescue organisms infected by these pathogens [18,26,27].

We tested the hypothesis that meropenem could be used in combination with Katrice-16, even for carbapenem-resistant bacteria, establishing that the combination could be useful to combat *K. pneumoniae* ST16. This finding represents a promising alternative to more efficiently treat bacterial isolates resistant to carbapenems. Similar results have been obtained combining meropenem (16–256 mg/L) with low concentrations of the myovirus KARL-1 (MOI 10^−1^–10^−7^), with significantly decreased MDR *A. baumannii* growth, demonstrating that our finding can also be applied to other gram negatives [28].

Katrice-16 additionally exhibited good anti-biofilm activity, especially at 12-h post-treatment (Figure 4(A)). We believe that the superior results obtained at 12-h versus 24-h treatment are related to the age of the biofilm since it is known that phages have higher efficiency in infecting cells with high metabolic activity, exactly the opposite of what is found in biofilms [29,30]. Although we only tested Katrice-16 anti-biofilm activity at 37 °C, we believe that at RT, the cocktail should remain effective since the bacteriophages demonstrated good activity at RT [25]. This is important in the case of *K. pneumoniae* ST16 since this microorganism has a great capacity to persist in hospital settings and on abiotic surfaces and as such, cause complex prosthetic joint infections [31].

## Conclusion

As new reports emerge indicating the spread of MDR *K. pneumoniae* ST16 [5,32], we have demonstrated that phage-based approaches are an attractive way to combat these micro-organisms due to their low cost, their good *in vitro* and *in vivo* activity and to their effectiveness in human body fluids. The rapidity of this approach is in stark contrast to the time taken to discover and evaluate an effective new antibiotic, and there is therefore great potential in using this approach to counteract emerging MDR pathogens such as *K. pneumoniae* ST16.

## Supplementary Material

Supplemental Material
