# Supplementary figures and images for: Effective phage cocktail to combat the rising incidence of extensively drug-resistant Klebsiella pneumoniae sequence type 16
Source: Emerg Microbes Infect. 2022 Apr 7;11(1):1015–23. doi: 10.1080/22221751.2022.2051752 (PMC9004492; doi:10.1080/22221751.2022.2051752)

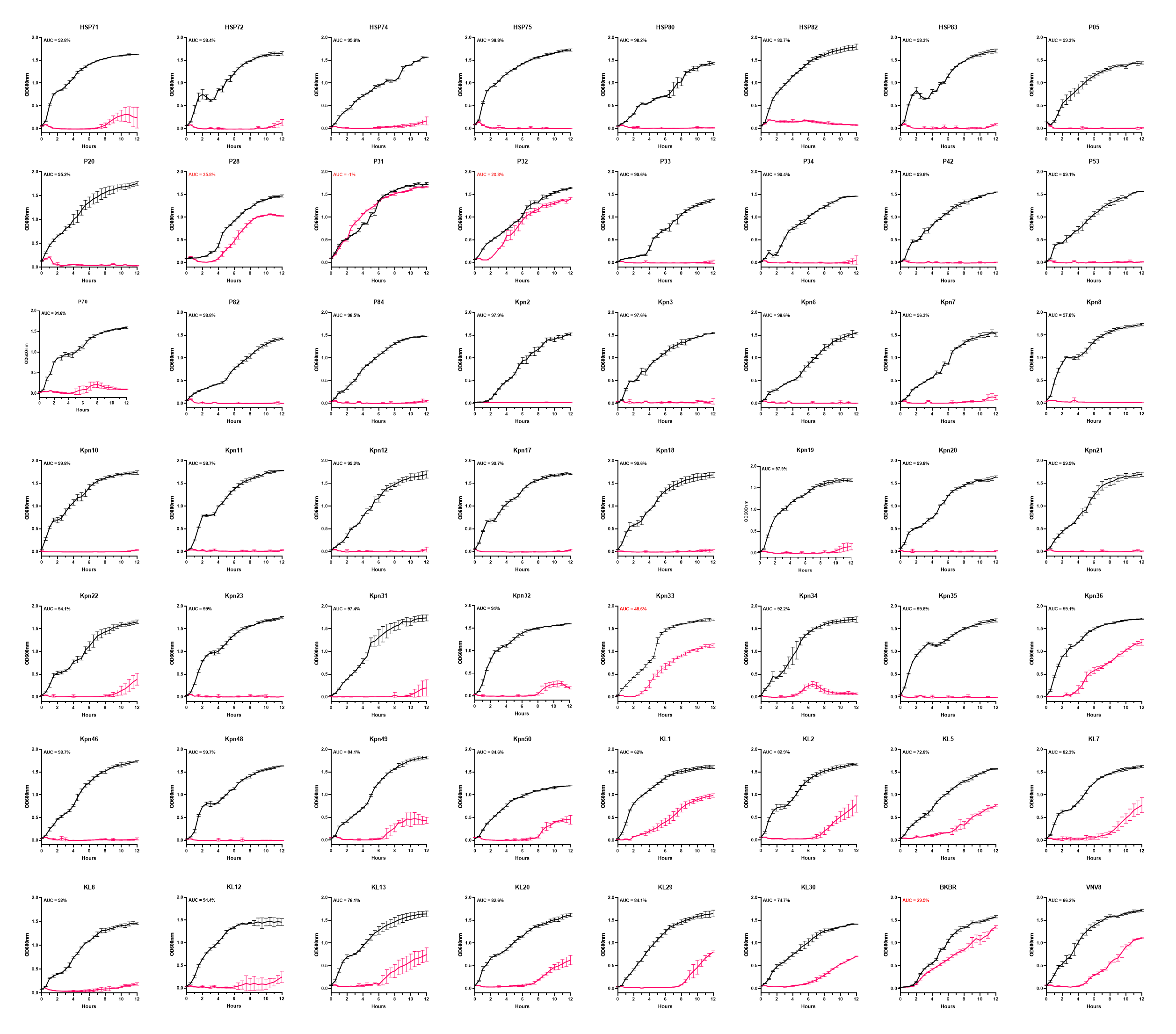

Supplement: Supplemental Material [file TEMI_A_2051752_SM7828.zip › Suppl files/Figure S1.tif]

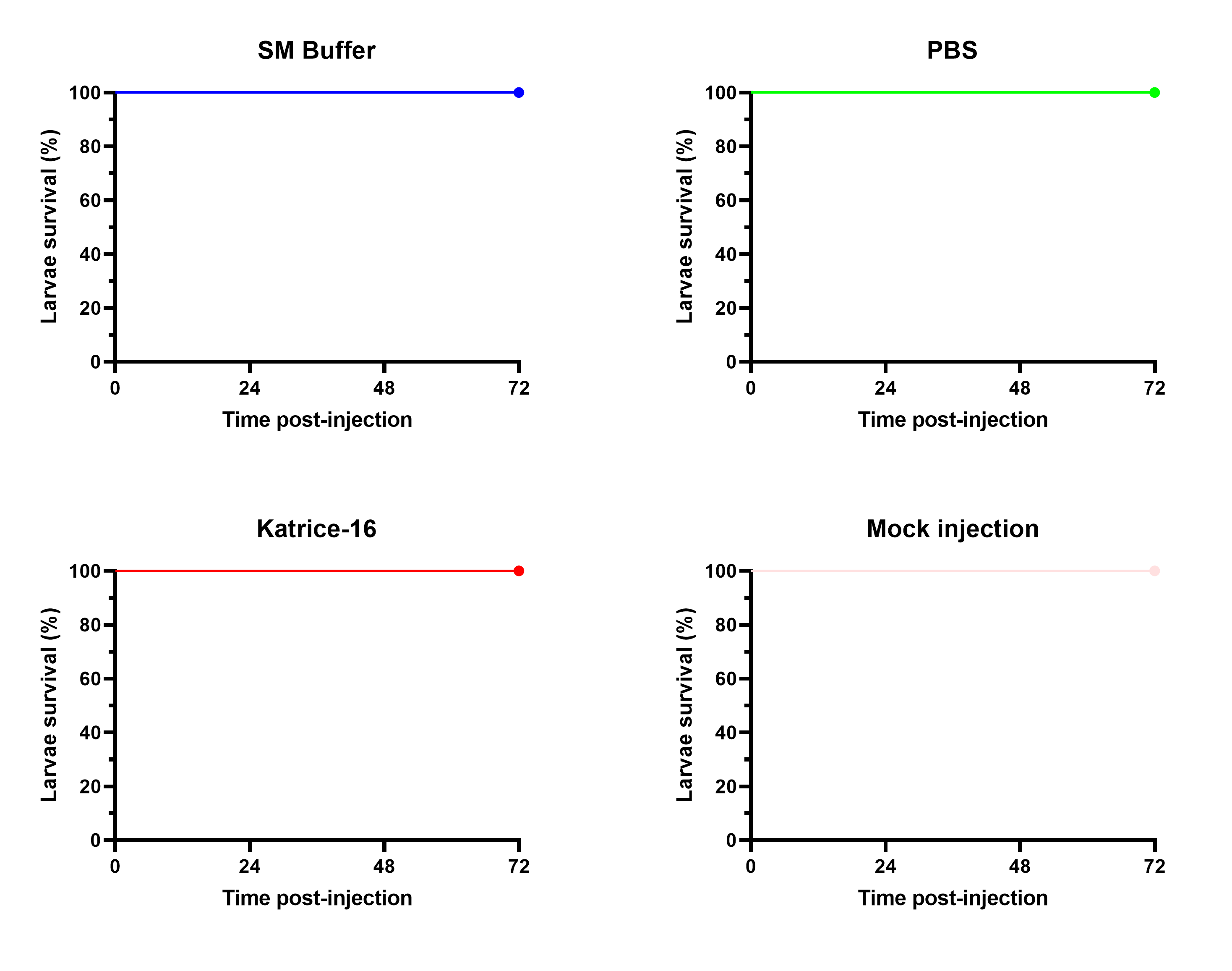

Supplement: Supplemental Material [file TEMI_A_2051752_SM7828.zip › Suppl files/Figure S2.tif]
